# Prosthetic aortic graft replacement of the ascending thoracic aorta alters biomechanics of the native descending aorta as assessed by transthoracic echocardiography

**DOI:** 10.1371/journal.pone.0230208

**Published:** 2020-03-12

**Authors:** Maria C. Palumbo, Lisa Q. Rong, Jiwon Kim, Pedram Navid, Razia Sultana, Jonathan Butcher, Alberto Redaelli, Mary J. Roman, Richard B. Devereux, Leonard N. Girardi, Mario F. L. Gaudino, Jonathan W. Weinsaft

**Affiliations:** 1 Departments of Cardiothoracic Surgery, Weill Cornell Medicine, New York, New York, United States of America; 2 Department of Electronics, Information and Bioengineering, Politecnico di Milano, Milan, Italy; 3 Department of Anesthesiology, Weill Cornell Medicine, New York, New York, United States of America; 4 Department of Medicine (Cardiology), Weill Cornell Medicine, New York, New York, United States of America; 5 Department of Biomedical Engineering, Cornell University, Ithaca, New York, United States of America; Universita degli Studi di Roma La Sapienza, ITALY

## Abstract

**Introduction:**

In patients with ascending aortic (AA) aneurysms, prosthetic graft replacement yields benefit but risk for complications in the descending aorta persists. Longitudinal impact of AA grafts on native descending aortic physiology is poorly understood.

**Methods:**

Transthoracic echocardiograms (echo) in patients undergoing AA elective surgical grafting were analyzed: Descending aortic deformation indices included global circumferential strain (GCS), time to peak (TTP) strain, and fractional area change (FAC). Computed tomography (CT) was used to assess aortic wall thickness and calcification.

**Results:**

46 patients undergoing AA grafting were studied; 65% had congenital or genetically-associated AA (30% bicuspid valve, 22% Marfan, 13% other): After grafting (6.4±7.5 months), native descending aortic distension increased, irrespective of whether assessed based on circumferential strain or area-based methods (both p<0.001). Increased distensibility paralleled altered kinetics, as evidenced by decreased time to peak strain (p = 0.01) and increased velocity (p = 0.002). Augmented distensibility and flow velocity occurred despite similar pre- and post-graft blood pressure and medications (all p = NS), and was independent of pre-surgical aortic regurgitation or change in left ventricular stroke volume (both p = NS). Magnitude of change in GCS and FAC was 5–10 fold greater among patients with congenital or genetically associated AA vs. degenerative AA (p<0.001), paralleling larger descending aortic size, greater wall thickness, and higher prevalence of calcific atherosclerotic plaque in the degenerative group (all p<0.05). In multivariate analysis, congenital/genetically associated AA etiology conferred a 4-fold increment in magnitude of augmented native descending aortic strain after proximal grafting (B = 4.19 [CI 1.6, 6.8]; p = 0.002) independent of age and descending aortic size.

**Conclusions:**

Prosthetic graft replacement of the ascending aorta increases magnitude and rapidity of distal aortic distension. Graft effects are greatest with congenital or genetically associated AA, providing a potential mechanism for increased energy transmission to the native descending aorta and adverse post-surgical aortic remodeling.

## Introduction

Prosthetic graft replacement is a well-established interventional therapy for patients with ascending thoracic aortic aneurysms (AA), in whom it provides potential lifesaving benefits and is recommended by consensus guidelines [1, 2]. While graft replacement eliminates risk for dilatation or dissection in surgically replaced regions, event risk persists in non-grafted areas–especially in patients with genetically associated aortopathies [3–7]. Nearly 50% of type B dissections in patients with Marfan syndrome occur in context of prior prophylactic graft surgery [5, 6]. Patients with bicuspid aortic valve are also at increased risk for recurrent clinical events, including re-operation after initial aortic valve replacement and/or prophylactic graft surgery [7, 8]. Given the clinical seriousness of such events, improved mechanistic insight into reasons for adverse changes in aortic physiology after graft implantation is of substantial importance.

One reason for heightened risk following prosthetic graft surgery may be due to altered vascular tissue properties of the native aorta. An added factor may stem from the impact of grafts on aortic physiology. Prosthetic grafts differ from the native aorta with respect to geometry and distensibility [9–12], and thus provide a stiff conduit to propagate high velocity flow into distal (non-grafted) segments. Consistent with this, prior studies by our group have shown ascending aortic grafts to acutely increase energy transmission to the native descending aorta, resulting in increased descending aortic distension as measured via intra-operative transesophageal echocardiography [13]. However, it remains uncertain whether such changes persist post-operatively in ambulatory patients (without modulatory effects of cardiac anesthesia), or whether magnitude of prosthetic graft-induced alterations in native aortic distension varies between patients with and without congenital or genetically associated aortopathies.

This study examined temporal changes in descending aortic mechanics among patients undergoing prosthetic graft replacement of the ascending aorta. To do so, transthoracic echocardiography (echo) was used to quantify descending aortic distension (strain) and flow pre- and post-operatively, together with input variables including left ventricular function and aortic size. Goals were to (1) determine impact of AA graft implantation on descending aortic distension post-operatively; and (2) identify pre-operative clinical, hemodynamic, and imaging variables associated with graft-induced effects on the native descending aorta.

## Materials and methods

### Study population

This entailed a retrospective review of pre-existing (imaging, clinical) data; informed consent for study participation was not obtained given the retrospective nature of this protocol. The study protocol was approved by the Weill Cornell Institutional Review Board, which approved analysis of pre-existing data for research purposes, approved query of institutional databases to identify eligible patients for this study (prior to data de-identification), and waived the requirement for informed consent. A pre-designated study investigator (JWW) oversaw access to patient identifiers for data query purposes prior to data de-identification for image/research analyses.

The population comprised patients who underwent surgical prosthetic graft replacement of the AA, in whom transthoracic echo was performed pre- and post-operatively. Patients were included based on availability of echo datasets inclusive of descending aortic images of adequate quality to assess distension pre- and (within two years) post-operatively. CT (available in 74%) was used to further test whether descending aortic wall thickness modified graft response. To examine the primary impact of prosthetic grafts on descending aortic physiology, patients with concomitant conditions known to affect aortic distension (i.e. dissection, severe aortic regurgitation or stenosis) were excluded.

Patients underwent prosthetic graft surgery and imaging at New York Presbyterian Hospital/Weill Cornell Medicine (New York, NY): Aortic reconstruction was performed using polyethylene terephthalate (Dacron) grafts. Demographic indices were compiled via review of electronic medical records. AA etiology (congenital/genetic, degenerative) and diagnostic categories were classified based on review of clinical/records and images by a dedicated study investigator (JK) blinded to strain analyses.

### Image acquisition

#### Echocardiography

Transthoracic echoes were performed in accordance with consensus (ACC/AHA, ESC) guidelines [14] using commercial equipment (Philips: iE33, EPIQ7 [Andover, MA]). Exams included short axis views of the mid-descending aorta, which was typically assessed using left ventricular (LV) long (e.g. 3 chamber) or short axis datasets. Additional data included pulsed and continuous wave Doppler to assess aortic regurgitation; pulsed wave Doppler was also used to measure peak flow velocity in the proximal descending aorta.

#### Computed tomography

Chest CT imaging was performed in accordance with standard clinical protocols. The imaging field of view varied based on body habitus, with adjustments made to encompass the thoracic aorta. Images were acquired using both ECG gated and non-gated algorithms. In 81% of exams, iodinated contrast was intravenously infused in conjunction with image acquisition with contrast timing tailored to opacify the thoracic aorta.

### Image analysis

#### Aortic strain

Aortic size and deformation were assessed on echo using short axis images (perpendicular to longitudinal plane of mid descending aorta). Strain analyses were performed using commercial software (TomTec [Unterschleissheim, Germany]). In brief, multiple (~10) seed points were placed equidistantly throughout the circumference of the aorta. Automated speckle tracking was then performed such that seed points were tracked throughout the cardiac cycle. Automated tracking analyses were visually inspected: If deemed suboptimal (discordant with visualized aortic contours), seed points were manually adjusted to optimize vessel tracking. Consistent with prior studies by our group and others [15–17], the aorta was partitioned into equidistant segments for which segmental results were averaged to derive the following indices:

- Global Circumferential Strain (GCS)–peak circumferential deformation of the aortic wall between systole and diastole (measured as the relative (%) difference between these two time points; [end-systole–end-diastole] / end-diastole * 100).
- Pulse Pressure Adjusted GCS–GCS normalized for pulse pressure (systolic–diastolic blood pressure), the latter of which was based on brachial measurements at time of echo [18].
- Time to peak (TTP) strain–calculated as the time interval between initial LV systolic contraction (R wave onset) and maximal circumferential aortic strain.

**Fig 1:**
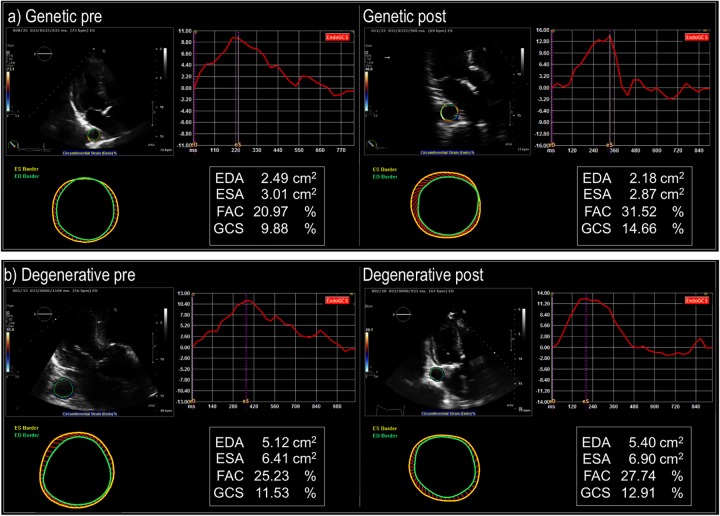
Representative examples of descending aortic strain assessed pre- and post- ascending aortic graft implantation in patients with congenital or genetically associated AA associated (top) and degenerative (bottom) AA. Circumferential strain over the entire cardiac cycle is represented by the red curve (upper right panel) from which systolic and diastolic frames were used to compute global circumferential strain (GCS); corresponding end-diastolic and end-systolic border delineation are represented by green and yellow circles (lower left). Note increased circumferential strain (Δ = 4.78) in a patient with congenitally associated aortic aneurysm (bicuspid aortic valve), despite similar pre-operative strain (Δ = 1.38) in a patient with a degenerative aortic aneurysm.

Fig 1 provides representative examples of aortic strain analyses performed using echo exams acquired pre- and post-proximal aortic grafting.

Aortic analyses also included quantification of end-systolic luminal area (ESA) and end-diastolic area (EDA) as well as corresponding linear diameters. Aortic area measurements were used to calculate fractional area change (FAC = [ESA–EDA]/EDA). In addition, aortic distensibility (AD) was calculated via an established equation (AD = [ESA–EDA] / [EDA x PP]), for which PP represents pulse pressure, and EDA and ESA the area of the descending aorta at end systole and end diastole [19].

#### Aortic regurgitation and LV remodeling

Aortic regurgitation (AR) was assessed based on 2D and Doppler indices of severity, including jet width, vena contracta, pressure half-time, and calculated regurgitant volume/ fraction: Aggregate data was used to assess AR severity, which was graded in accordance with consensus guidelines [20].

LV chamber size and function were quantified on echo based on standardized linear dimensions measured in parasternal long axis orientation, concordant with established methods validated in prior research [21, 22]. LV mass was quantified using anteroseptal and posterior wall thickness and LV diastolic dimension, in accordance with necropsy validated methods [23].

#### Aortic vessel wall characteristics

Aortic wall thickness was measured on CT using (double oblique) reformatted short axis images in the mid descending thoracic aorta. CT datasets were also reviewed for presence of calcific atherosclerotic plaque in the aortic arch and descending thoracic aorta, which was graded in a binary (present/absent) manner.

### Statistical analyses

Comparisons between groups were made using Student’s t-test (expressed as mean ± standard deviation) for continuous variables. Categorical variables were compared using chi-square or Fisher’s exact test (when < 5 expected outcomes per cell). Bivariate correlation coefficients were used to evaluate associations between continuous variables. Univariable and multivariate modeling was performed via linear regression. Reproducibility of GCS was assessed based on mean difference and limits of agreement (mean ± 1.96 standard deviation) between repeated measurements, as well as intra-class correlation coefficients (ICC). Statistical calculations were performed using SPSS 22.0 (SPSS Inc. [Chicago, IL]). Two-sided p<0.05 was considered indicative of statistical significance.

## Results

### Population characteristics

The population comprised 46 patients with AA aneurysms who underwent elective surgical prosthetic graft repair. Aneurysms were attributable to congenital or genetically associated conditions in over half (63% [n = 30]) patients, including 30% (n = 14) with bicuspid aortic valve, 22% (n = 10) with Marfan syndrome and 13% (n = 6) with other conditions. In the remaining 37% (n = 16) of patients, AA was deemed sporadic (degenerative) in etiology.

Table 1 reports baseline clinical and imaging characteristics of study participants, including comparisons between patients with congenital or genetically associated and degenerative AA. As shown, patients with congenital or genetically associated AA were younger and less likely to have systemic hypertension or coronary artery disease (CAD) (p<0.05), but were similar with respect to medication regimen. Regarding imaging, descending aortic size was smaller in patients with congenital or genetically associated AA (p<0.001), but both LV systolic function and severity of (≤ severe) aortic regurgitation were similar between groups (p = NS).

**Table 1:** Population characteristics.

	Overall (n = 46)	Congenital / Genetic (n = 30)	Degenerative (n = 16)	p
**Clinical**				
Age [years]	52 ± 18	44 ± 15	68 ± 13	**<0.001**
Body Surface Area [m^2^]	2.0 ± 0.3	2.0 ± 0.3	1.9 ± 0.2	0.07
Male gender	29 (63%)	21 (70%)	8 (50%)	0.18
AA Etiology				
Bicuspid Aortic Valve	14 (30%)	14 (47%)	-	-
Marfan Syndrome	10 (22%)	10 (33%)	-	-
Other*	6 (13%)	6 (20%)	-	-
Coronary Artery Disease	5 (11%)	0 (0%)	5 (31%)	**0.003**
Cardiovascular Medications				
Beta-blocker	20 (44%)	11 (37%)	9 (56%)	0.20
ACE-Inhibitor	5 (11%)	2 (7%)	3 (19%)	0.33
Angiotensin Receptor Blocker	14 (30%)	10 (33%)	4 (25%)	0.56
Hypertension	19 (41%)	8 (27%)	11 (69%)	**0.006**
Diabetes	3 (7%)	1 (3%)	2 (12%)	0.27
Tobacco	3 (7%)	2 (7%)	1 (6%)	1.00
Concomitant Aortic Valve Replacement	20 (44%)	10 (33%)	10 (63%)	0.11
**Imaging**				
LV End-Diastolic Volume [ml/m^2^]	78.0 ± 22.8	76.7 ± 12.2	80.6 ± 36.3	0.69
LV End-Systolic Volume [ml/m^2^]	34.1 ± 20.2	31.3 ± 8.6	41.2 ± 35.4	0.35
LV Stroke Volume [ml/m^2^]	44.9 ± 10.0	45.8 ± 8.8	43.1 ± 12.2	0.41
LV Cardiac Output [l/min]	5.6 ± 1.4	5.9 ± 1.4	5.0 ± 1.3	0.08
LV Ejection Fraction [%]	58.4 ± 10.6	59.5 ± 8.3	55.7 ± 14.9	0.30
LV Mass [g/m^2^]	95.3 ± 29.9	87.9 ± 20.9	110.0 ± 39.5	0.06
Aortic Regurgitation [mild, mild-moderate, moderate]	7 (15%) / 7 (15%) / 9 (20%)	6 (20%) / 4 (13%) / 6 (20%)	1 (6%) / 3 (19%) / 3 (19%)	0.82
Ascending Thoracic Aortic Size [cm]	4.8 ± 0.6	4.7 ± 0.6	5.0 ± 0.7	0.16
Descending Thoracic Aortic Size [cm]	2.3 ± 0.4	2.2 ± 0.3	2.6 ± 0.4	**0.001**

*Loeys-Dietz syndrome (n = 2), vascular Ehlers-Danlos syndrome (n = 1), indeterminate (n = 3)

### Graft-associated alterations in native aortic distention

Echo was performed at median [inter-quartile range] intervals of 1.9 [0.6–4.5] and 2.4 [0.7–12.4] months pre- and post-operatively. Table 2 reports changes in native descending aortic biomechanics between pre- and post-operative time points, as assessed by distension and flow indices. As shown, descending aortic distension increased post-operatively, irrespective of whether assessed based on circumferential strain or area-based methods (both p<0.001). Increased distention paralleled altered kinetics, as evidenced by decreased time to peak strain (p<0.05).

**Table 2:** Descending aortic biomechanics before and after ascending aortic graft replacement.

	Overall			Congenital/Genetic			Degenerative		
	Pre	Post	p	Pre	Post	p	Pre	Post	p
Global circumferential strain [%]	8.6 ± 3.2	11.7 ± 4.6	**<0.001**	8.7 ± 2.9	13.3 ± 4.1	**<0.001**	8.4 ± 3.7	8.7 ± 4.1	0.65
TTP [ms]	328.1± 63.6	290.3 ± 72.6	**0.01**	333.4 ± 53.5	307.7 ± 67.5	0.13	318.2 ± 80.1	257.6 ± 72.6	**0.01**
TTP/RR [%]	35.6 ± 8.3	31.8 ± 9.3	**0.02**	35.9 ± 9.2	32.2 ± 8.3	0.07	34.8 ± 6.8	31.0 ± 11.2	0.19
Δ Area/TTP [cm^2^/s]	2.4 ± 1.1	3.7 ± 2.2	**<0.001**	2.2 ± 0.9	3.4 ± 1.5	**<0.001**	2.8 ± 1.3	4.1 ± 3.1	**0.04**
PP corrected strain (CAS/PP)	19.2 ± 7.8	26.4 ± 13.8	**<0.001**	19.6 ± 7.0	28.1 ± 9.6	**<0.001**	18.2 ± 9.6	23.1 ± 20.0	0.27
Distensibility [10^−3^ mmHg^-1^]	4.1 ± 1.7	5.6 ± 2.9	**<0.001**	4.1 ± 1.5	6.0 ± 2.2	**<0.001**	3.9 ± 2.1	4.8 ± 4.0	0.27
Δ Area [cm^2^]	0.8 ± 0.3	1.0 ± 0.4	**<0.001**	0.7 ± 0.3	1.0 ± 0.4	**<0.001**	0.9 ± 0.4	0.9 ± 0.5	0.70
Fractional Area Change [%]	18.3 ± 7.0	24.9 ± 10.4	**<0.001**	18.4 ± 6.5	28.5 ± 9.4	**<0.001**	18.0 ± 8.1	18.2 ± 9.0	0.87

TTP = time to peak strain; PP = pulse pressure; CAS = global peak circumferential aortic strain

**Fig 2:**
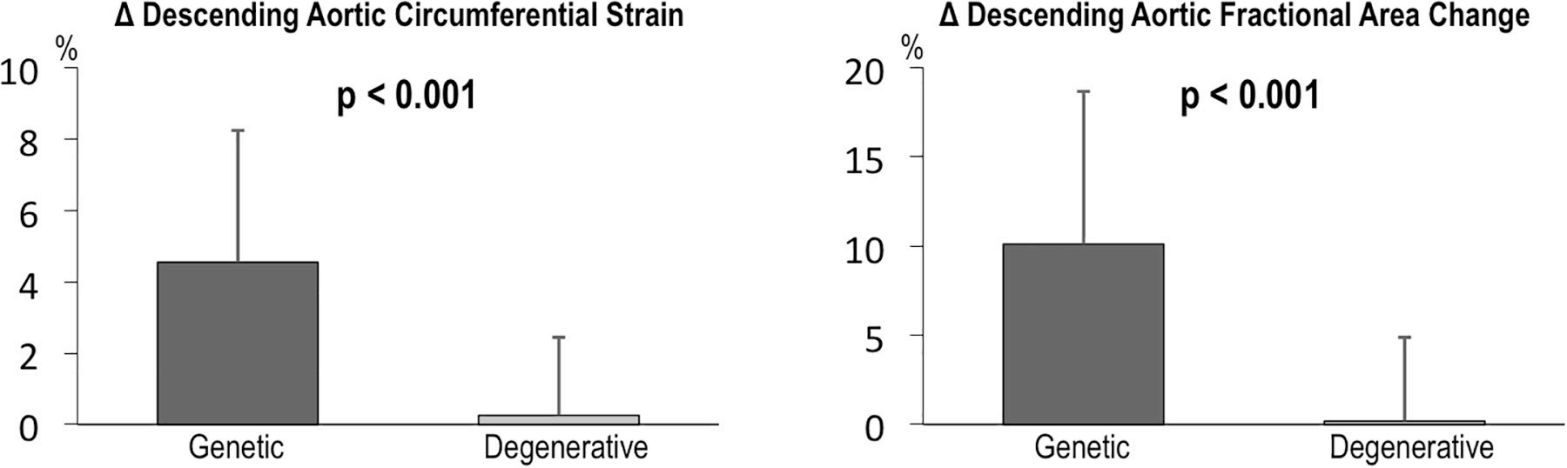
Differences in post- vs. pre-operative descending aortic strain (left) and fractional area change in patients with congenital or genetically associated and degenerative AA (data shown as mean±standard deviation). Note that both distension indices similarly increased to a greater extent among patients with congenital or genetically associated AA.

Alterations in descending aortic distensibility primarily occurred in patients with congenital or genetically associated aneurysms: As shown in Table 2, sub-group analyses demonstrated GCS and FAC to increase post-operatively (p<0.001) among congenital or genetic AA aneurysm patients; among patients with degenerative aneurysms only kinetic indices were altered significantly (p<0.05) whereas primary strain or area-based indices were not (p = NS). Fig 2 illustrates that magnitude of change in GCS and FAC was 5–10 fold greater among patients with congenital or genetically associated compared to degenerative AA (p<0.001). Of note, magnitude of augmentation in aortic distensibility (2.1 ± 1.8 vs. 1.6 ± 2.2 [10-3mmHg-1]) and strain indices were numerically greater among patients with Marfan/genetically associated AA compared to BAV although differences between groups did not achieve significance (all p>0.10) in context of limited sample size (n = 16 Marfan/genetically associated AA, n = 14 BAV). Regarding geometry, descending aortic size was similar between patients with Marfan syndrome compared to those with BAV, irrespective of whether assessed on pre-operative (2.2±0.4 vs. 2.3±0.3 cm, p = 0.33) or post-operative echo (2.3±0.3 cm vs. 2.3±0.3, p = 0.91).

Of note, time to peak strain decreased across the study population (p = 0.01), reflecting similar patterns in patients with congenital or genetically associated and degenerative AA aneurysms. Similarly, pulsed wave Doppler derived velocity (assessable in 59% of patients) increased post-operatively (89.5 ± 29.9 vs. 106.0 ± 36.3 cm/s, p = 0.002). Despite differences in imaging-evidenced temporal indices, non-invasive pulse pressure was similar pre- and post-operatively (48.8 ± 13.8 vs. 50.0 ± 14.5mmHg, p = 0.64), possibly reflecting imprecision of brachial arterial blood pressure measurements.

Reproducibility of strain indices was tested in 15 random exams (33% of study population), among whom descending aortic GCS strain yielded good intra-observer (intra-class correlation [ICC]: 0.91 [0.77–0.97], p<0.001| **Δ** = 0.39, limits of agreement [LOA] = -3.40, 2.62), and inter-observer reproducibility (ICC: 0.90 [0.46–0.97], p<0.001 | **Δ** = -1.16, LOA = -3.61, 1.30).

### Hemodynamics and aortic remodeling

**Fig 3:**
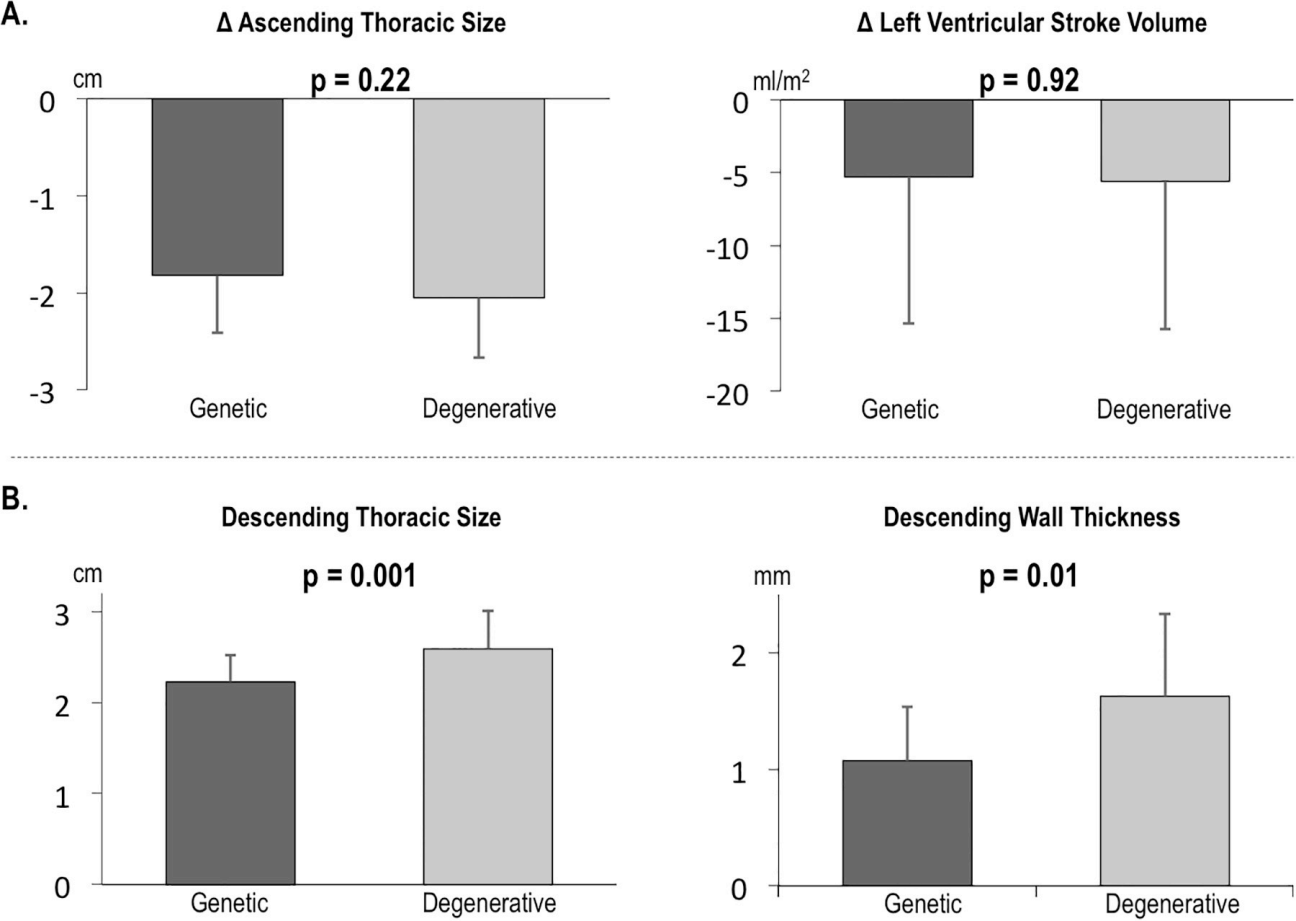
Aortic morphology and left ventricular performance indices between AA subtypes. (A) Note similar changes in ascending aortic size and LV stroke volume between groups, without significant differences either pre- or post-operatively. (B) Conversely, both pre-operative descending aortic size and wall thickness were greater among patients with degenerative AA. Data shown as mean ± standard deviation.

To elucidate causality of graft-induced changes in native aortic distension, hemodynamic and aortic remodeling indices were assessed pre-and post-operatively. As shown in Table 3, blood pressure and heart rate were similar between time points (p = NS). Regarding LV performance, stroke volume declined (p = 0.001)–a finding likely attributable to post-surgical improvement in aortic regurgitation. However, as shown in Fig 3A, magnitude of changes in both LV stroke volume and ascending aortic size were similar between patients with degenerative and congenital or genetically associated AA (both p = NS).

**Table 3:** Hemodynamic and imaging characteristics in relation prosthetic graft surgery.

	Pre	Post	p	Pre	Post	p	Pre	Post	p
***Hemodynamics***									
Heart Rate	63.4 ± 11.3	64.7 ± 11.4	0.49	63.4 ± 12.6	64.5 ± 12.4	0.64	63.6 ± 7.8	65.3 ± 8.9	0.56
Systolic Blood Pressure [mmHg]	120.6 ± 16.3	118.8 ± 18.4	0.48	117.5 ± 12.5	114.9 ± 14.9	0.38	127.1 ± 21.4	126.8 ± 22.7	0.95
Diastolic Blood Pressure [mmHg]	71.8 ± 9.3	68.8 ± 10.4	0.12	71.6 ± 8.3	67.1 ± 10.6	0.07	72.3 ± 11.4	72.4 ± 9.2	0.98
Pulse Pressure [mmHg]	48.8 ± 13.8	50.0 ± 14.5	0.64	45.9 ± 10.6	47.8 ± 9.2	0.39	54.9 ± 17.8	54.4 ± 21.7	0.95
***Imaging***									
Aortic End Diastolic Area [cm^2^]	4.3 ± 1.4	4.1 ± 1.4	0.23	3.9 ± 1.0	3.7 ± 0.9	0.09	5.1 ± 1.6	5.1 ± 1.7	0.97
Aortic End Systolic Area [cm^2^]	5.0 ± 1.6	5.1 ± 1.6	0.70	4.6 ± 1.2	4.7 ± 1.1	0.60	5.9 ± 1.8	5.9 ± 1.9	0.96
Ascending Thoracic Aorta [cm]	4.8 ± 0.6	2.9 ± 0.2	**<0.001**	4.7 ± 0.6	2.9 ± 0.3	**<0.001**	5.0 ± 0.7	2.9 ± 0.2	**<0.001**
Descending Thoracic Aorta [cm]	2.3 ± 0.4	2.4 ± 0.4	0.16	2.2 ± 0.3	2.3 ± 0.3	0.24	2.6 ± 0.4	2.6 ± 0.4	0.43
LV End-Diastolic Volume [ml/m^2^]	76.0 ± 22.8	68.5 ± 19.3	**0.01**	76.8 ± 12.4	70.8 ± 17.6	0.07	74.0 ± 39.5	62.6 ± 23.1	0.08
LV End-Systolic Volume [ml/m^2^]	32.5 ± 20.0	30.9 ± 14.1	0.42	30.6 ± 8.5	30.0 ± 10.0	0.77	40.3 ± 41.1	35.2 ± 24.3	0.51
LV Ejection Fraction [%]	59.4 ± 9.1	56.8 ± 10.0	0.06	60.1 ± 7.5	57.9 ± 7.9	0.15	56.8 ± 13.7	52.6 ± 15.2	0.26
LV Stroke Volume [ml/ m^2^]	44.4 ± 9.5	39.0 ± 11.0	**0.001**	45.8 ± 8.8	40.5 ± 10.7	**0.007**	41.5 ± 10.5	35.9 ± 11.4	0.051

LV = left ventricle

Regarding aortic morphology, Fig 3B demonstrates that pre-operative descending aortic size was larger among patients with degenerative AA aneurysms; results were near equivalent on post-operative echo (both p = 0.001). CT (available in 74% of patients) demonstrated similar differences in descending aortic size (2.5 ± 0.3 vs. 2.9 ± 0.6 cm, p = 0.09), while also demonstrating patients with degenerative AA to have higher wall thickness (1.1 ± 0.5 vs. 1.6 ± 0.7 mm, p = 0.01) and higher prevalence of calcific atherosclerotic plaque in the descending aorta (82% vs. 4%, p<0.001).

### Predictors of graft-induced increments in native descending aortic strain

Regression analysis was employed to discern relative impact of pre-operative clinical and imaging variables on post-operative increments in native aortic strain. As shown in Table 4, congenital or genetic AA etiology (p<0.001) and younger age (p = 0.03) were the sole clinical parameters associated with increased GCS. Regarding imaging, descending aortic size was negatively associated with increased GCS (p = 0.01). Of note, change in descending aortic strain was not associated with follow-up interval between graft surgery and post procedural echo.

**Table 4:** Univariate regression analysis for change in decending aortic strain.

	B	95% Confidence Interval	p
**Clinical**			
Congenital/Genetic AA Etiology	4.3	(2.3, 6.3)	**<0.001**
Age [per 10 year decrement]	0.67	(0.07, 1.3)	**0.03**
Body Surface Area [m^2^]	-1.72	(-6.0, 2.5)	0.42
Male gender	0.13	(-2.2, 2.5)	0.91
Known CAD	-1.90	(-5.6, 1.7)	0.30
Hypertension	-1.07	(-3.4, 1.2)	0.36
Diabetes mellitus	-1.20	(-5.8, 3.4)	0.60
Tobacco use	0.66	(-4.0, 5.3)	0.77
Concomitant Aortic Valve Replacement	-0.70	(-3.0, 1.6)	0.54
Follow-up Interval	-0.11	(-0.27, 0.04)	0.13
**Medications**			
Beta blocker	-0.19	(-2.5, 2.1)	0.87
Angiotensin Receptor Blocker	1.55	(-2.1, 5.2)	0.40
ACE-Inhibitor	1.87	(-1.9, 5.6)	0.32
**Imaging**			
LV Stroke Volume [ml/m^2^]	-0.01	(-0.1, 0.1)	0.90
Aortic Regurgitation [grade]	0.05	(-0.6, 0.7)	0.89
Ascending Thoracic Aortic Size [cm]	-0.30	(-2.1, 1.5)	0.74
Descending Thoracic Aortic Size [cm]	-3.96	(-6.8, -1.1)	**0.01**
**Change (Δ) vs. baseline**			
Δ LV End-Diastolic Volume [ml/m^2^]	0.02	(-0.05, 0.09)	0.64
Δ LV End-Systolic Volume [ml/m^2^]	-0.02	(-0.12, 0.08)	0.71
Δ LV Stroke Volume [ml/m^2^]	0.06	(-0.06, 0.17)	0.33
Δ Ascending Thoracic Aortic Size [cm]	-0.23	(-2.1, 1.7)	0.81
Δ Descending Thoracic Aortic Size [cm]	-0.30	(-5.47, 4.87)	0.91
Δ Heart Rate	-0.04	(-0.13, 0.04)	0.28
Δ Systolic Blood Pressure [mmHg]	-0.002	(-0.07, 0.07)	0.96
Δ Diastolic Blood Pressure [mmHg]	-0.01	(-0.10, 0.08)	0.81
Δ Pulse Pressure [mmHg]	0.005	(-0.07, 0.08)	0.89

As shown in Table 5, multivariable modeling demonstrated congenital or genetic AA etiology to be independently associated with greater post-operative increments in descending aortic GCS: Results demonstrated congenital or genetic etiology to confer over a 4-fold increment in magnitude of augmented strain (p = 0.002), with a lesser trend for descending aortic size (p = 0.12) and a non-significant association with age (p = 0.36).

**Table 5:** Multivariate regression analysis for change in decending aortic strain.

*Model r*^*2*^ = *0*.*263*, *p = 0*.*001*	B	95% Confidence Interval	p
Congenital/Genetic AA Etiology	4.19	(1.6, 6.8)	**0.002**
Age [per 10 year decrement]	-0.37	(-1.2, 0.4)	0.36
Descending Thoracic Aortic Size	-2.66	(-6.0, 0.72)	0.12

## Discussion

This study demonstrates that prosthetic grafting of the ascending thoracic aorta produces sustained alterations in native descending aortic biomechanics, which are most marked among patients with congenital or genetically associated AA. Aortic distention–whether assessed based on GCS or FAC–increased among congenital or genetically associated AA patients, but was unchanged among patients with degenerative AA. Increased distention was strongly associated with differences in aortic morphology, as evidenced by smaller descending aortic caliber, lesser wall thickness, and lower prevalence of aortic calcification among patients with congenital or genetically associated AA.

Our current findings build upon prior research by our group, in which intraoperative trans-esophageal echocardiography was employed to demonstrate that circumferential deformation and distensibility of the distal native aorta increased significantly acutely after prosthetic replacement of the ascending aorta [15]. Additionally, our results provide mechanistic insight into prior clinical studies demonstrating that risk for adverse aortic remodeling (e.g. dilation, dissection) of the descending aorta persists after proximal grafting, and that such risks are greatest in patients with genetically associated aortopathies. Among 1991 patients with genetic AA aneurysms followed in the NIH-sponsored GenTAC registry, over half (52%) of all dissections occurred in patients who had undergone prior prophylactic AA graft repair and 71% occurred in the distal aortic arch or descending aorta [5]. Similarly, among a cohort of 600 patients with Marfan syndrome, den Hartog et al reported that prophylactic aortic surgery was associated with a >2-fold increase in risk for subsequent type B aortic dissection [24]. Even in absence of dissection, longitudinal studies have reported an association between prosthetic grafting and adverse remodeling: Among a cohort of 189 Marfan syndrome patients without dissection, surgical grafting of the ascending aorta was associated with a 4-fold increase in subsequent dilation of the distal aorta, independent of age or gender [25]. While studies have generally shown risk to be greatest in patients with Marfan syndrome, it should also be noted that other genetically associated aortopathies augment risk for both incident aortic dissection as well as post-operative events following proximal aortic repair [7, 26]. For example, among patients undergoing surgical aortic root reconstruction, Svensson et al reported that bicuspid valve morphology conferred increased risk for aortic reoperation [7]. While it is well-established that native aortic tissue properties influence prognosis, our findings support the notion that augmented risk for clinical events following prosthetic grafting may in part be attributable to graft induced alterations in distal aortic flow, wall stress, and distension.

**Fig 4:**
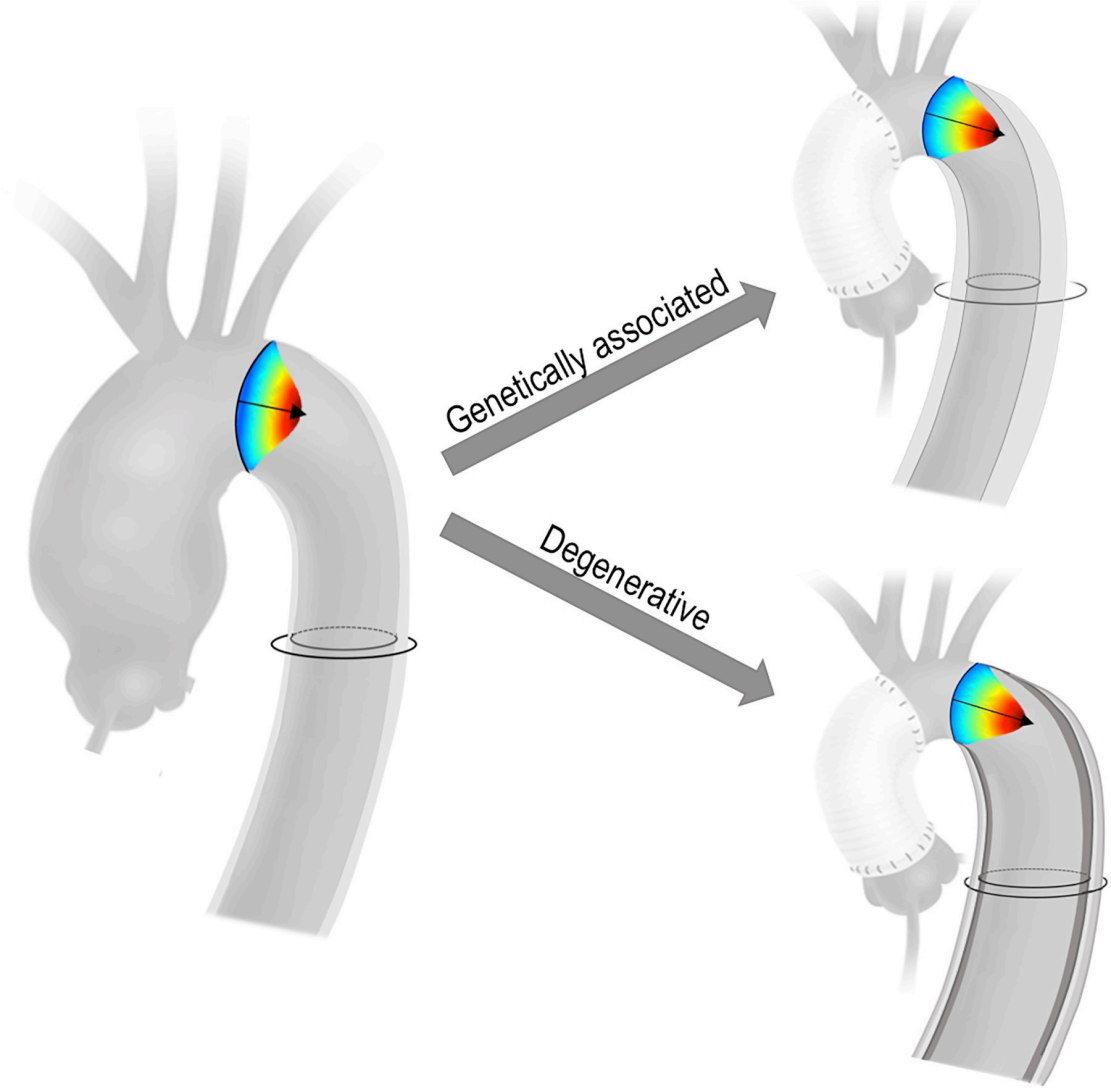
Proposed mechanisms underlying differential response of congenital or genetically associated and degenerative AA to prosthetic aortic grafting. Whereas both AA subtypes manifest increased flow velocity in response to implantation of a relatively non-compliant ascending graft, lesser downstream instrinsic aortic stiffness in patients with congenital or genetic AA results in increased descending aortic distension.

Our results are consistent with prior research employing computational simulation and animal models [10, 27–29]. In an ex-vivo study for which explanted porcine aortas were studied under mock circulatory conditions, Scharfschwerdt et al demonstrated that prosthetic graft replacement of the ascending aorta increased distal aortic systolic pressure and wall tension [27]. In a computational fluid dynamics study, Vardoulis et al reported that prosthetic grafting of the ascending aorta augmented amplitude and slope of forward pressure propagation [10]. Similar results have been shown using in-vivo data in a porcine model, in which impaired proximal aortic compliance (consistent with effects of prosthetic grafting) increased pulse pressure amplitude and decreased pressure recovery in the native descending aorta [29]. These data are consistent with findings of our study, which demonstrated that increased distension was accompanied by increased descending aortic flow velocity and decreased time to peak strain–temporal indices which would be expected to be altered in the context of non-compliant grafts. Regarding differential response between degenerative and congenital or genetically associated AA, our data–which demonstrate increased distention to be paralleled by lesser wall thickness and less frequent presence of calcific plaque–are consistent with broader concepts in vascular physiology for which atherosclerotic plaque has been shown to reduce aortic compliance [30, 31]. Our findings support a central mechanism whereby prosthetic graft replacement of the ascending thoracic aorta produces similar hemodynamic modifications (including amplitude and velocity of systolic forward flow) irrespective of AA etiology, but that consequences on the aortic wall vary due to differences in downstream native aortic tissue properties (Fig 4).

It is important to contextualize our findings with prior studies that have shown aortic stiffness to be increased in patients with Marfan syndrome, [32, 33] and reported increased stiffness to confer risk for aortic dissection [24, 34]. Our finding of increased descending aortic distention after proximal aortic graft implantation supports the notion that graft induced changes in aortic physiology are sufficient to overcome intrinsic tissue properties in patients with Marfan syndrome. It is also possible that Marfan patients generally experience an increase in descending aortic distention after ascending aortic prosthetic graft surgery, but that a continuum exists such that individuals with maximal pre-procedural aortic stiffness are at greatest risk for subsequent clinical events. As pre-procedural aortic compliance would be expected to modify graft induced alterations in biomechanics, we believe that our findings support the need for future larger scale research to test whether pre-operative vessel wall properties and graft induced aortic distention (increased strain) are synergistic with respect to their impact on risk for adverse aortic remodeling and post-operative aortic dissection.

Several limitations should be noted. First, it is important to recognize that our results demonstrate relatively short term changes in native aortic flow and distensibility in the months following preventive graft repair of dilated AAs: Longer term research is necessary to elucidate whether graft-induced effects are sustained, and whether such effects vary based on diagnosis (i.e. Marfan vs. bicuspid valve) or vascular tissue properties. Second, it is important to recognize that whereas current analyses demonstrated good reproducibility of GCS within our dataset, further research is warranted to establish reproducibility across vendor platforms and in larger epidemiologic cohorts. It should also be noted that our study examined native aortic distension as an endpoint rather than actual clinical events, and that our study entailed longitudinal imaging comparisons in patients undergoing surgery: Although lack of a (non-surgical) control group prohibited us from fully excluding disease evolution as a cause for change in descending aortic distensibility, descending aortic size was similar (p = NS) before and after surgical grafting—supporting the notion that changes in aortic strain were not attributable to localized aortic remodeling (dilation) as would be expected in the context of disease progression. Moreover, our current results build upon prior work by our group in which intra-operative transesophageal echo data demonstrated that descending aortic strain increased near immediately after graft implantation–supporting the notion that graft effects on aortic biomechanics were primarily responsible for our observed longitudinal changes in strain on serial transthoracic echo. Last, it is important to recognize that whereas our data showed native descending aortic distension to similarly increase in patients with genetic and BAV-associated AA, clinical studies have shown that risk for dissection after graft surgery is elevated in Marfan syndrome patients whereas risks in patients with BAV is predominantly (if not exclusively) limited to recurrent aneurysmal dilation [8]. In this context, it is possible that these two groups ultimately respond differently to altered distending forces induced by a prosthetic graft, resulting in differences in clinical outcomes. Further longitudinal research in larger cohorts is needed to test these concepts [3–8].

In conclusion, findings of this study suggest that prosthetic graft replacement of the ascending aorta increases magnitude and early timing of distal aortic distension in patients with congenital or genetically associated AA. Future studies are warranted to test whether post-operative changes in native aortic distensibility varies based on extent of grafting or genetic etiology of AA, and whether graft-induced effects are modifiable based on graft design or predictive of long-term event risk.

## Supporting information

S1 File Minimal data set. A de-identified data set encompassing key variables used for analysis has been included.
(XLSX)

## References

[1] Hiratzka LF, Bakris GL, Beckman JA, Bersin RM, Carr VF, Casey DE Jr., et al 2010 ACCF/AHA/AATS/ACR/ASA/SCA/SCAI/SIR/STS/SVM guidelines for the diagnosis and management of patients with Thoracic Aortic Disease: a report of the American College of Cardiology Foundation/American Heart Association Task Force on Practice Guidelines, American Association for Thoracic Surgery, American College of Radiology, American Stroke Association, Society of Cardiovascular Anesthesiologists, Society for Cardiovascular Angiography and Interventions, Society of Interventional Radiology, Society of Thoracic Surgeons, and Society for Vascular Medicine. Circulation. 2010;121(13):e266–369. 10.1161/CIR.0b013e3181d4739e . 20233780

[2] Erbel R, Aboyans V, Boileau C, Bossone E, Bartolomeo RD, Eggebrecht H, et al 2014 ESC Guidelines on the diagnosis and treatment of aortic diseases: Document covering acute and chronic aortic diseases of the thoracic and abdominal aorta of the adult. The Task Force for the Diagnosis and Treatment of Aortic Diseases of the European Society of Cardiology (ESC). Eur Heart J. 2014;35(41):2873–926. 10.1093/eurheartj/ehu281 . 25173340

[3] Gaudino M, Girardi LN, Rahouma M, Leonard JR, Di Franco A, Lau C, et al Editor’s Choice—Aortic Re-operation After Replacement of the Proximal Aorta: A Systematic Review and Meta-Analysis. Eur J Vasc Endovasc Surg. 2018;56(4):515–23. Epub 2018/07/25. 10.1016/j.ejvs.2018.06.038 . 30037741

[4] Song HK, Kindem M, Bavaria JE, Dietz HC, Milewicz DM, Devereux RB, et al Long-term implications of emergency versus elective proximal aortic surgery in patients with Marfan syndrome in the Genetically Triggered Thoracic Aortic Aneurysms and Cardiovascular Conditions Consortium Registry. J Thorac Cardiovasc Surg. 2012;143(2):282–6. Epub 2011/11/23. 10.1016/j.jtcvs.2011.10.024 . 22104675 PMC3260411

[5] Weinsaft JW, Devereux RB, Preiss LR, Feher A, Roman MJ, Basson CT, et al Aortic Dissection in Patients With Genetically Mediated Aneurysms: Incidence and Predictors in the GenTAC Registry. J Am Coll Cardiol. 2016;67(23):2744–54. Epub 2016/06/11. 10.1016/j.jacc.2016.03.570 . 27282895 PMC5040186

[6] Roman MJ, Devereux RB, Preiss LR, Asch FM, Eagle KA, Holmes KW, et al Associations of Age and Sex With Marfan Phenotype: The National Heart, Lung, and Blood Institute GenTAC (Genetically Triggered Thoracic Aortic Aneurysms and Cardiovascular Conditions) Registry. Circ Cardiovasc Genet. 2017;10(3). Epub 2017/06/11. 10.1161/CIRCGENETICS.116.001647 . 28600386 PMC5500868

[7] Svensson LG, Pillai ST, Rajeswaran J, Desai MY, Griffin B, Grimm R, et al Long-term survival, valve durability, and reoperation for 4 aortic root procedures combined with ascending aorta replacement. J Thorac Cardiovasc Surg. 2016;151(3):764–74.e4. 10.1016/j.jtcvs.2015.10.113 . 26778214 PMC5125725

[8] Itagaki S, Chikwe JP, Chiang YP, Egorova NN, Adams DH. Long-Term Risk for Aortic Complications After Aortic Valve Replacement in Patients With Bicuspid Aortic Valve Versus Marfan Syndrome. J Am Coll Cardiol. 2015;65(22):2363–9. 10.1016/j.jacc.2015.03.575 . 26046728

[9] Tremblay D, Zigras T, Cartier R, Leduc L, Butany J, Mongrain R, et al A comparison of mechanical properties of materials used in aortic arch reconstruction. Ann Thorac Surg. 2009;88(5):1484–91. 10.1016/j.athoracsur.2009.07.023 . 19853098

[10] Vardoulis O, Coppens E, Martin B, Reymond P, Tozzi P, Stergiopulos N. Impact of aortic grafts on arterial pressure: a computational fluid dynamics study. Eur J Vasc Endovasc Surg. 2011;42(5):704–10. 10.1016/j.ejvs.2011.08.006 . 21889370

[11] Miyawaki F, How TV, Annis D. Effect of compliance mismatch on flow disturbances in a model of an arterial graft replacement. Med Biol Eng Comput. 1990;28(5):457–64. 10.1007/bf02441969 . 2277546

[12] Stewart SF, Lyman DJ. Effects of a vascular graft/natural artery compliance mismatch on pulsatile flow. J Biomech. 1992;25(3):297–310. 10.1016/0021-9290(92)90027-x . 1564063

[13] Rong LQ, Palumbo MC, Rahouma MM, Meineri M, Arguelles GR, Kim J, et al Immediate of Impact Prosthetic Graft Replacement of the Ascending Aorta on Circumferential Strain in the Descending Aorta. Eur J Vasc Endovasc Surg. 2019 10.1016/j.ejvs.2019.05.003 . 31445862

[14] Lang RM, Badano LP, Mor-Avi V, Afilalo J, Armstrong A, Ernande L, et al Recommendations for cardiac chamber quantification by echocardiography in adults: an update from the American Society of Echocardiography and the European Association of Cardiovascular Imaging. Eur Heart J Cardiovasc Imaging. 2015;16(3):233–70. Epub 2015/02/26. 10.1093/ehjci/jev014 . 25712077

[15] Rong LQ, Palumbo MC, Rahouma M, Meineri M, Arguelles GR, Kim J, et al Immediate Impact of Prosthetic Graft Replacement of the Ascending Aorta on Circumferential Strain in the Descending Aorta. European Journal of Vascular and Endovascular Surgery 2019;(in press). 10.1016/j.ejvs.2019.05.003 31445862

[16] Alreshidan M, Shahmansouri N, Chung J, Lash V, Emmott A, Leask RL, et al Obtaining the biomechanical behavior of ascending aortic aneurysm via the use of novel speckle tracking echocardiography. J Thorac Cardiovasc Surg. 2017;153(4):781–8. Epub 2017/01/18. 10.1016/j.jtcvs.2016.11.056 . 28094007

[17] Cruz C, Pinho T, Sousa C, Dias CC, Silva Cardoso J, Maciel MJ. Ascending aorta in tetralogy of Fallot: Beyond echocardiographic dimensions. Echocardiography. 2018;35(9):1362–9. Epub 2018/06/15. 10.1111/echo.14046 . 29900594

[18] Oishi Y, Mizuguchi Y, Miyoshi H, Iuchi A, Nagase N, Oki T. A novel approach to assess aortic stiffness related to changes in aging using a two-dimensional strain imaging. Echocardiography. 2008;25(9):941–5. Epub 2008/09/06. 10.1111/j.1540-8175.2008.00725.x . 18771548

[19] Voges I, Jerosch-Herold M, Hedderich J, Pardun E, Hart C, Gabbert DD, et al Normal values of aortic dimensions, distensibility, and pulse wave velocity in children and young adults: a cross-sectional study. J Cardiovasc Magn Reson. 2012;14:77 Epub 2012/11/16. 10.1186/1532-429X-14-77 . 23151055 PMC3514112

[20] Zoghbi WA, Adams D, Bonow RO, Enriquez-Sarano M, Foster E, Grayburn PA, et al Recommendations for Noninvasive Evaluation of Native Valvular Regurgitation: A Report from the American Society of Echocardiography Developed in Collaboration with the Society for Cardiovascular Magnetic Resonance. J Am Soc Echocardiogr. 2017;30(4):303–71. 10.1016/j.echo.2017.01.007 . 28314623

[21] Palmieri V, Dahlof B, DeQuattro V, Sharpe N, Bella JN, de Simone G, et al Reliability of echocardiographic assessment of left ventricular structure and function: the PRESERVE study. Prospective Randomized Study Evaluating Regression of Ventricular Enlargement. J Am Coll Cardiol. 1999;34(5):1625–32. 10.1016/s0735-1097(99)00396-4 . 10551715

[22] Kim J, Di Franco A, Seoane T, Srinivasan A, Kampaktsis PN, Geevarghese A, et al Right Ventricular Dysfunction Impairs Effort Tolerance Independent of Left Ventricular Function Among Patients Undergoing Exercise Stress Myocardial Perfusion Imaging. Circulation Cardiovascular imaging. 2016;9(11). 10.1161/CIRCIMAGING.116.005115 27903538 PMC5137788

[23] Devereux RB, Alonso DR, Lutas EM, Gottlieb GJ, Campo E, Sachs I, et al Echocardiographic assessment of left ventricular hypertrophy: comparison to necropsy findings. Am J Cardiol. 1986;57(6):450–8. 10.1016/0002-9149(86)90771-x 2936235

[24] den Hartog AW, Franken R, Zwinderman AH, Timmermans J, Scholte AJ, van den Berg MP, et al The risk for type B aortic dissection in Marfan syndrome. J Am Coll Cardiol. 2015;65(3):246–54. 10.1016/j.jacc.2014.10.050 . 25614422

[25] Engelfriet PM, Boersma E, Tijssen JG, Bouma BJ, Mulder BJ. Beyond the root: dilatation of the distal aorta in Marfan’s syndrome. Heart. 2006;92(9):1238–43. 10.1136/hrt.2005.081638 . 16488927 PMC1861183

[26] Michelena HI, Khanna AD, Mahoney D, Margaryan E, Topilsky Y, Suri RM, et al Incidence of aortic complications in patients with bicuspid aortic valves. JAMA. 2011;306(10):1104–12. 10.1001/jama.2011.1286 . 21917581

[27] Scharfschwerdt M, Sievers HH, Greggersen J, Hanke T, Misfeld M. Prosthetic replacement of the ascending aorta increases wall tension in the residual aorta. Ann Thorac Surg. 2007;83(3):954–7. 10.1016/j.athoracsur.2006.10.056 . 17307439

[28] Bauernschmitt R, Schulz S, Schwarzhaupt A, Kiencke U, Vahl CF, Lange R, et al Simulation of arterial hemodynamics after partial prosthetic replacement of the aorta. Ann Thorac Surg. 1999;67(3):676–82. 10.1016/s0003-4975(99)00046-6 . 10215210

[29] Ioannou CV, Stergiopulos N, Katsamouris AN, Startchik I, Kalangos A, Licker MJ, et al Hemodynamics induced after acute reduction of proximal thoracic aorta compliance. Eur J Vasc Endovasc Surg. 2003;26(2):195–204. 10.1053/ejvs.2002.1917 . 12917838

[30] Siegel E, Thai WE, Techasith T, Major G, Szymonifka J, Tawakol A, et al Aortic distensibility and its relationship to coronary and thoracic atherosclerosis plaque and morphology by MDCT: insights from the ROMICAT Trial. Int J Cardiol. 2013;167(4):1616–21. 10.1016/j.ijcard.2012.04.107 . 22578738 PMC3419779

[31] Al-Mallah MH, Nasir K, Katz R, Takasu J, Lima JA, Bluemke DA, et al Thoracic aortic distensibility and thoracic aortic calcium (from the Multi-Ethnic Study of Atherosclerosis [MESA]). Am J Cardiol. 2010;106(4):575–80. 10.1016/j.amjcard.2010.03.074 . 20691319 PMC4228943

[32] Singh P, Almarzooq Z, Codell NCF, Wang Y, Roman MJ, Devereux RB, et al Cine-CMR partial voxel segmentation demonstrates increased aortic stiffness among patients with Marfan syndrome. J Thorac Dis. 2017;9(Suppl 4):S239–S45. 10.21037/jtd.2017.04.02 . 28540066 PMC5422665

[33] Yan J, Lehsau AC, Sauer B, Pieper B, Mohamed SA. Comparison of biomechanical properties in ascending aortic aneurysms of patients with congenital bicuspid aortic valve and Marfan syndrome. Int J Cardiol. 2019;278:65–9. 10.1016/j.ijcard.2018.11.102 . 30527531

[34] Rooprai J, Boodhwani M, Beauchesne L, Chan KL, Dennie C, Nagpal S, et al Thoracic Aortic Aneurysm Growth in Bicuspid Aortic Valve Patients: Role of Aortic Stiffness and Pulsatile Hemodynamics. J Am Heart Assoc. 2019;8(8):e010885 10.1161/JAHA.118.010885 . 30966855 PMC6507195

